# Causal effects of homocysteine levels on the components of sarcopenia: A two-sample mendelian randomization study

**DOI:** 10.3389/fgene.2022.1051047

**Published:** 2022-11-22

**Authors:** Hongwei Yu, Gan Luo, Tianwei Sun, Qiong Tang

**Affiliations:** ^1^ School of Medicine, Nankai University, Tianjin, China; ^2^ Graduate School of Tianjin Medical University, Tianjin, China; ^3^ Department of Spinal Surgery, Tian-jin Union Medical Centre, Nankai University People’s Hospital, Tianjin, China; ^4^ Department of Respiratory Medicine, Tian-jin Union Medical Centre, Nankai University People’s Hospital, Tianjin, China

**Keywords:** homocysteine, grip strength, walking pace, appendicular lean mass, mendelian randomization

## Abstract

**Background:** Currently, it is unclear whether there is a causal association between genetically predicted plasma homocysteine (Hcy) levels and the risk of sarcopenia. We performed a Mendelian randomization (MR) study to assess the association between circulating Hcy levels and the components [grip strength, walking pace, and appendicular lean mass (ALM)] of sarcopenia.

**Methods:** Independent single nucleotide polymorphisms (SNPs) significantly associated with plasma Hcy levels served as instrumental variables. Summary-level data regarding the components of sarcopenia. Were obtained from the UK Biobank. Inverse variance weighted (IVW) as the primary method was used for Mendelian randomization (MR) analysis. We also use four models, weighted median, MR-Egger regression, Maximum likelihood, and Penalised weighted median, as supplementary methods to IVW. The MR-Egger intercept test, Cochran’s Q test, and “leave-one-out” sensitivity analysis were performed to evaluate the horizontal pleiotropy, heterogeneities, and stability of the causal association between Hcy levels and the components of sarcopenia.

**Results:** The IVW-MR analysis suggested significant negative associations of increased plasma Hcy levels with grip strength (right: effect = −0.036, SE = 0.032, *p* = 5.53E-4; left: effect = −0.045, SE = 0.010, *p* = 1.45E-5), walking pace (effect = −0.038, SE = 0.011, *p* = 3.18E-4), and ALM (effect = −0.058, 0.013, *p* = 1.03E-5). However, there were no significant associations of decreased plasma Hcy levels with grip strength (right: effect = 0.005, SE = 0.021, *p* = 0.82; left: effect = −0.006, SE = 0.014, *p* = 0.64), walking pace (effect = 0.01, 0.020, *p* = 0.61), or ALM (effect = -0.034, SE = 0.018, *p* = 0.06).The accuracy and robustness of these findings were confirmed by sensitivity tests.

**Conclusion:** Increased circulating Hcy levels were associated with lower grip strength, slower walking pace, and decreased ALM.

## 1 Introduction

Sarcopenia is defined as an age-related loss of muscle mass and function (Cruz-Jentoft et al., 2010). In 2018, the European Working Group on Sarcopenia in Older People (EWGSOP) redefined sarcopenia to emphasize that muscle function (muscle strength and physical performance) is the most important component of sarcopenia, as muscle strength is a better predictor of poor outcomes than muscle mass (Cruz-Jentoft et al., 2019). Worldwide, as many as 50 million people have sarcopenia, with sarcopenia most prevalent in elderly individuals. With aging of the global population, the prevalence of sarcopenia is expected to increase (Fernandes et al., 2022); the number of people with sarcopenia is predicted to reach 500 million by 2050 (Bhasin et al., 2020). Sarcopenia leads to decreased muscle strength and flexibility, fatigue, and increased risks of falls, fractures, physical disability, and death (Álvarez-Bustos et al., 2022; Yu et al., 2022); these symptoms and risks severely reduce the quality of life of patients and impose a greater burden on families and society (Álvarez-Bustos et al., 2022). An increasing number of studies have investigated sarcopenia, identifying possible causative factors of nutritional dysfunction (Pagano et al., 2018), altered endocrine function (Fielding et al., 2011), and a systemic inflammatory response (Dolan et al., 2019). However, the causative factors of sarcopenia are complex and diverse; thus, they merit further investigation.

Homocysteine (Hcy) is a sulfur-containing amino acid, that is, an intermediate product of methionine and cysteine metabolism (Selhub, 1999). A variety of factors can lead to increased plasma levels of Hcy, resulting in hyperhomocysteinemia. Increased Hcy levels can damage cells, tissues and organs and affect the body (Yoo et al., 2022). Clinical studies have found an association between plasma Hcy levels and risks of cardiovascular disease (Yuan et al., 2021), cerebrovascular disease (Spence and Hankey, 2022), kidney disease (Samson et al., 2022) and skeletal muscle system disease (Veeranki et al., 2015); thus, Hcy levels merit as much clinical monitoring as blood glucose, blood pressure and blood lipids. Epidemiological studies have shown an association between increased plasma Hcy levels and decreased muscle strength and physical function (Ter Borg et al., 2016; Lee et al., 2020). Observational studies have further expanded these associations of decreased muscle strength to include decreased muscle mass (McDermott et al., 2007; Choi et al., 2022). However, the findings of recent observational studies are somewhat contradictory. Cross-sectional and longitudinal studies by Granic et al. (2017) and Eguchi et al. (2021), respectively, did not find an association between Hcy levels and muscle function; similarly, long-term longitudinal studies by Elshorbagy et al. (2008) and Swart et al. (2016) did not find an association between Hcy levels and muscle mass. These conflicting findings may be because observational studies are inevitably subject to confounding factors and/or reverse causation. Furthermore, even if observational studies reveal consistent associations between Hcy levels and sarcopenia, it remains unclear whether Hcy levels are a causal factor for sarcopenia or a concomitant manifestation of sarcopenia due to a common causal factor. Randomized controlled trials (RCTs) are useful for controlling for confounding factors and directly inferring a causal relationship between variables (e.g., plasma Hcy levels and sarcopenia). However, in reality, RCTs are difficult to perform due to the expenses of recruiting human participants and obtaining materials, lengthy durations, and possible ethical constraints (Davies et al., 2018). Mendelian randomization (MR) studies complement RCTs; their design is based on the principle of random assignment of alleles at gamete formation and the use of the genotype as an instrumental variable to examine the intermediate phenotype and thus infer its causal association with the disease state. Hence, the effect estimates of MR studies are not influenced by confounding factors or reverse causal associations (Davies et al., 2018).

Muscle and bone, two important components of the locomotor system, are jointly regulated by a variety of physiological factors. The combination of sarcopenia and osteoporosis is referred to as dysmobility syndrome (Chen et al., 2021); this syndrome is one of the leading causes of falls, fractures, disability and even death in elderly individuals. Previous MR studies have identified that decreased plasma Hcy levels can causally increase bone mineral density (Wang et al., 2021). Here, we used MR analysis to investigate whether there is a causal association between increased or decreased plasma Hcy levels and the components of sarcopenia (grip strength, walking pace, and ALM), respectively. In addition, we used the MR-Egger intercept test, Cochran’s Q test, and“leave-one-out” sensitivity analysis to evaluate the horizontal pleiotropy, heterogeneities, and stability of the causal association between Hcy levels and the components of sarcopenia.

## 2 Methods

### 2.1 Study design

To ensure that reliable causal relationships are obtained, MR studies need to satisfy the following three core assumptions (Cruz-Jentoft et al., 2010, Pagoni et al., 2019) relevance, i.e., the instrumental variables are strongly correlated with the exposure factors; (Cruz-Jentoft et al., 2019); independence, i.e., the instrumental variables are not correlated with confounders of exposure and outcome; and (Fernandes et al., 2022) exclusion restriction, i.e., the instrumental variables can only affect the outcome through the exposure factors.

### 2.2 Data sources

Genetic variants associated with plasma Hcy levels were derived from the genome-wide association study (GWAS) meta-analysis published by van Meurs et al. (2013). This meta-analysis included nine cohorts of 44,147 individuals of European ancestry and identified 18 single nucleotide polymorphisms (SNPs) significantly associated with plasma Hcy levels. For more information on the above nine cohorts, please refer to the Supplementary Presentation. Regarding the sarcopenia components, grip strength is moderately correlated with strength in other parts of the body; thus, it can be used as a reliable substitute for whole-body strength measurements (Cruz-Jentoft et al., 2019). Walking pace is considered a fast, safe and highly reliable test for sarcopenia and is widely used in clinical practice (Cruz-Jentoft et al., 2019). Therefore, we selected grip strength and walking speed as the outcomes. GWAS data for both grip strength and walking pace were obtained from the UK Biobank (Cox, 2018). In brief, the UK Biobank is a large prospective cohort study that recruited over 500,000 participants (age range: 43%–79% and 54% female) in the UK from 2006 to 2010. Grip strength data (*n* = 461,089) were collected using the Jamar dynamometer, which is a validated and widely used method of measuring grip strength (Cox, 2018). Self-reported walking pace (*n* = 335,349) was obtained from answers to the question “How would you describe your usual walking speed?” The response options were “slow”, “steady/average”, and “fast”. Slow was defined as less than three miles per hour, steady/average was defined as between 3–4 miles per hour, and fast was defined as more than four miles per hour (Cox, 2018). Because direct measurement of muscle mass is usually not possible, lean body mass is considered a valid measure of muscle mass. ALM, the most commonly used muscle mass approximator in sarcopenia studies, is widely used in the EWGSOP (2) and Asian Working Group for Sarcopenia (AWGS) (Chen et al., 2020) diagnostic criteria for sarcopenia. We chose ALM rather than whole-body lean mass (WBLM) because ALM reduces the influence of the systemic water component, nonadipose soft tissue, cardiac muscle and vascular smooth muscle on the results (Cruz-Jentoft et al., 2019). ALM data were extracted from a GWAS by Pei et al. (2020) (*n* = 450,243), in which study samples were obtained from the UK Biobank. These ALM data were obtained by using bioelectric impedance analysis (BIA) to determine the sum of the deglutition masses of the arms and legs (Cox, 2018). The data sources for both exposure and outcome are summarized in Table 1.

**TABLE 1:** Data sources used for the MR analysis ALM:appendicular lean mass.

	Traits	Sample size	Number of SNPS	Population	Consortium or cohort study
Exposure	Plasma Homocysteine levels	44147	2,090,256	European	Van Meurs, et al./PMID:23824729
Outcomes	Grip strength (right)	461,089	9,851,867	European	UK Biobank (MRC IEU)/(https://doi.org/10.5523/bris.2fahpksont1zi26xosyamqo8rr)
	Grip strength (left)	461,026	9,851,867	European	UK Biobank (MRC IEU)/(https://doi.org/10.5523/bris.2fahpksont1zi26xosyamqo8rr)
	Walking pace	335,349	10,894,596	European	UK Biobank (Neale Lab)/(http://www.nealelab.is/uk-biobank)
	ALM	450243	18,071,518	European	Pei YF, et al./PMID:33097823

### 2.3 Extraction of instrumental variables

To satisfy assumption 1 (i.e., the relevance assumption), we selected SNPs from the initial 18 SNPs that were independently (R^2^ < 0.001, window size = 10,000 kb) and significantly associated with plasma Hcy levels at a genome-wide significance level (*p* < 5E−8) as potential instrumental variables (IVs). In addition, to ensure that the potential IVs had sufficient power to detect the causal influence of exposure on the outcomes, we calculated the F-statistics of the potential IVs using an online tool (https://sb452.shinyapps.io/overlap). IVs with F-statistics >10 were considered to have sufficiently robust estimation power to determine causal effects and were considered candidate IVs. To satisfy assumptions two and 3 (i.e., the independence and exclusion-restriction assumptions, respectively), we first identified confounders associated with plasma Hcy levels, muscle function and mass from three meta-analyses (Han et al., 2017; Shen et al., 2019; Gao et al., 2021), and then the PhenoScanner database (Version 2; http://www. phenoscanner.medschl.cam.ac.uk/) was used to examine the association of each candidate IV with the confounders. Candidate IVs were excluded if they had a significant association with outcomes or confounders (*p* < 5E-8). Once we identified the included IVs, we performed the first round of MR analysis. As the exact biological functions of many genetic variants remain unknown, we also used Mendelian randomization pleiotropy residual sum and outlier (MR-PRESSO) to identify and remove pleiotropic SNPs (Verbanck et al., 2018). If pleiotropic SNPs were identified by MR-PRESSO in the first round of analysis, we excluded them and before performing the second round of MR analysis.

### 2.4 Mendelian randomization analysis

The inverse variance-weighted Mendelian randomization (IVW-MR) method was the primary method used to assess causal effects between plasma Hcy levels and grip strength, walking pace, and ALM. IVW-MR uses a meta-analysis to combine Wald estimates for each SNP to obtain an overall estimate of the effect of exposure on outcomes (Burgess et al., 2016). IVW-MR can use both fixed- and random-effects models. In the present study, if significant heterogeneity was observed (*p* < 0.05), a random-effects IVW-MR model was applied. We also used MR-Egger (Bowden et al., 2015), weighted median (Bowden et al., 2016), maximum likelihood (Milligan, 2003), and penalized weighted median (Bowden et al., 2015) methods to complement and validate the results of the IVW-MR analysis. Detailed information regarding the above MR methods can be found elsewhere. If multiple comparisons were conducted, a Bonferroni-corrected *p* value < 0.0125 (0.05/4) was considered statistically significant. All analyses were performed using R version 4.1.1 with the two-sample MR package.

### 2.5 Sensitivity analysis

To further ensure the robustness of our MR estimates, we performed the following sensitivity analyses. First, Cochran’s Q test was used to quantify the heterogeneity among the genetic instruments (Pierce and Burgess, 2013). Second, the MR-Egger intercept test (Burgess and Thompson, 2017) and MR-PRESSO global test (Verbanck et al., 2018)were used to examine whether our MR analysis was affected by horizontal pleiotropy. Third, we performed a leave-one-out analysis to examine whether the overall estimates were disproportionately affected by specific SNPs.

## 3 Results

### 3.1 Single nucleotide polymorphisms selection and validation

Of the initial 18 SNPs, we excluded five (rs12134663, rs12921383, rs7422339, rs2851391, and rs957140) due to linkage disequilibrium. Additionally, rs1801133 was excluded due to its significant association with folic acid (P = 1E-28). The remaining 12 SNPs were categorized as IVs in our study (Table 2). Seven of these SNPs (rs9369898, rs154657, rs4660306, rs548987, rs1801222, rs12780845, and rs838133) were associated with increases in genetically predicted plasma levels of Hcy, and five SNPs (rs2275565, rs2251468, rs234709, rs7130284, and rs42648) were associated with decreases in genetically predicted plasma levels of Hcy. All 12 SNPs were valid (F > 10).

**TABLE 2:** Summary statistics for homocysteine-associated SNPs using as instrumental variables.

SNP	Chrr	Nearby gene	EA	OA	EAF	Beta	SE	*p* Value	F	Homocysteine levels
rs9369898	6	MUT	A	G	0.62	0.045	0.007	2.20E–10	42	unit increase
rs154657	16	DPEP1	A	G	0.47	0.096	0.007	1.70E–43	204	unit increase
rs4660306	1	MMACHC	T	C	0.33	0.043	0.007	2.30E–09	37	unit increase
rs548987	6	SLC17A3	C	G	0.13	0.06	0.01	1.10E–08	36	unit increase
rs1801222	10	CUBN	A	G	0.34	0.045	0.007	8.40E–10	41	unit increase
rs12780845	10	CUBN	A	G	0.65	0.053	0.009	7.80E–10	56	unit increase
rs838133	19	FUT2	A	G	0.45	0.042	0.007	7.50E–09	39	unit increase
rs2275565	1	MTR	G	T	0.21	−0.054	0.009	2.00E–10	43	unit decrease
rs2251468	12	HNF1A	C	A	0.65	−0.051	0.007	1.30E–12	53	unit decrease
rs234709	21	CBS	C	T	0.45	−0.072	0.007	3.90E–24	113	unit decrease
rs7130284	11	NOX4	T	C	0.07	−0.124	0.013	1.90E–20	89	unit decrease
rs42648	7	GTPB10	A	G	0.4	−0.039	0.007	2.00E–08	33	unit decrease

SNP: single-nucleotide polymorphism; Chr: chromosome; EA: effect allele; OA, other allele; EAF: effect allele frequency. F: F statistic; SE: standard error.

### 3.2 Associations of increased plasma hcy levels with grip strength, walking pace, and appendicular lean mass

**FIGURE 1:**
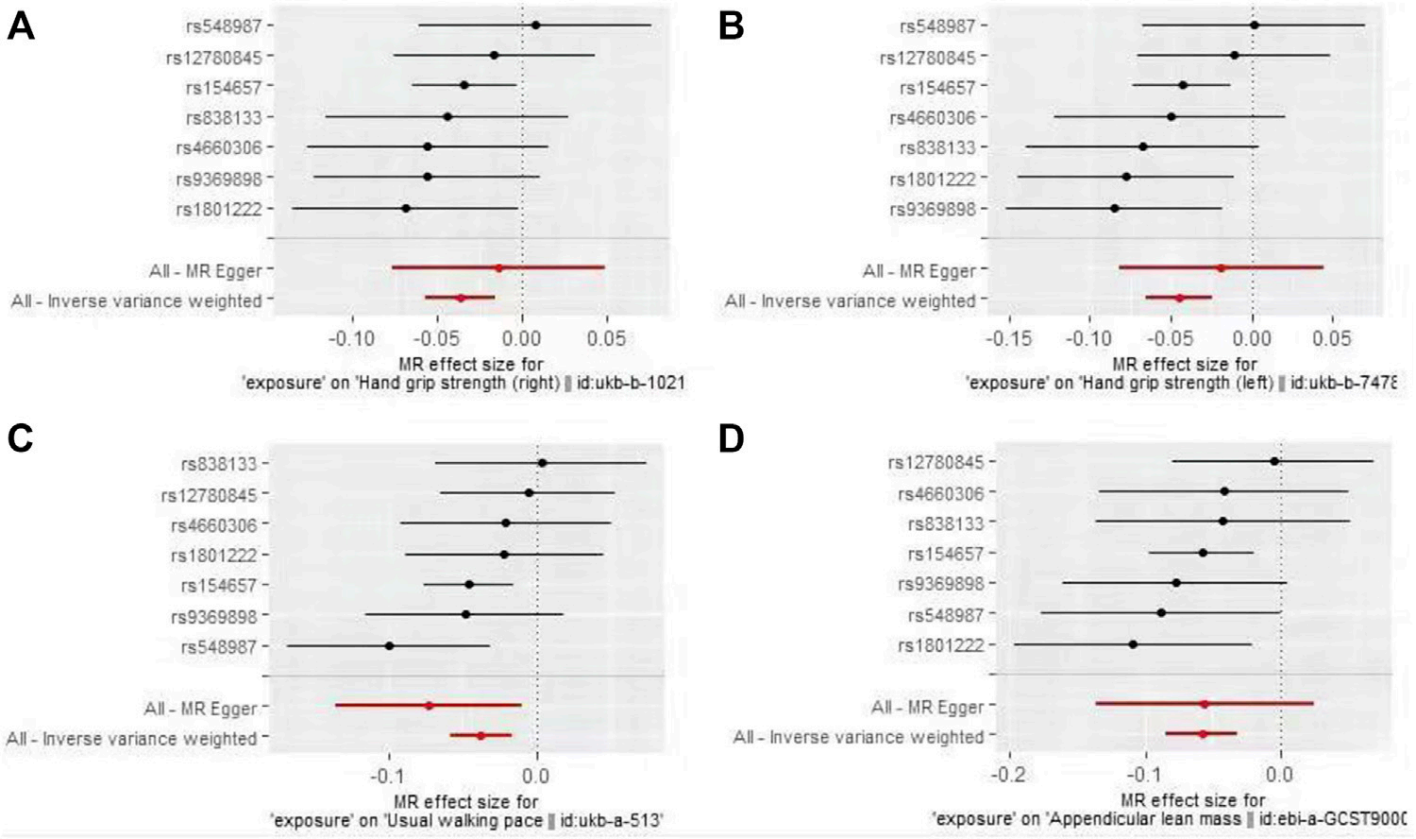
Forest plots for MR analyses of the causal effect of increased plasma homocysteine levels on Grip strength (right, **(A)**, Grip strength (left, **(B)**, Walking pace **(C)**, and ALM **(D)**.

**FIGURE 2:**
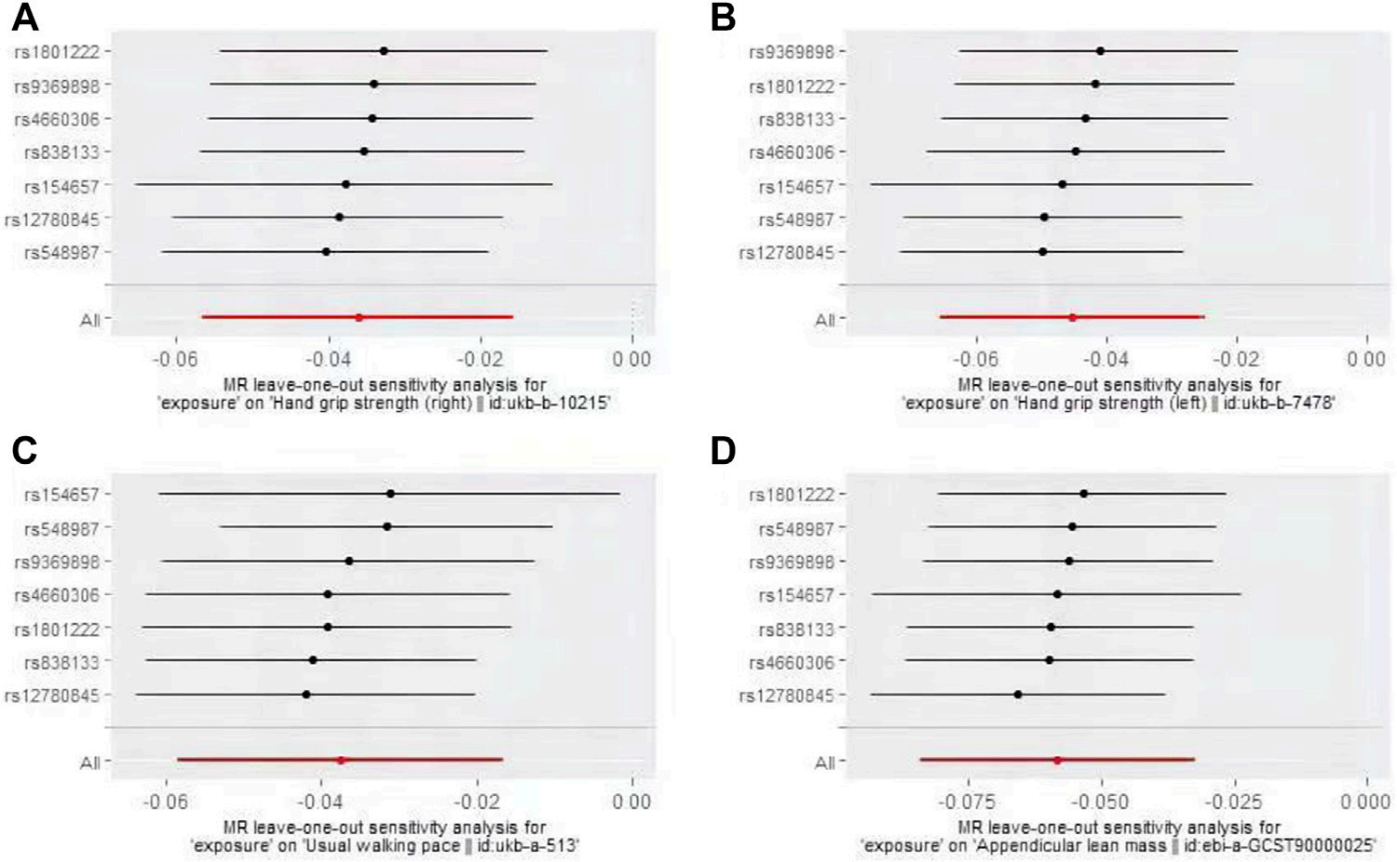
Plots of “leave-one-out”analyses MR analyses of the causal effect of increased plasma homocysteine levels on Grip strength (right, **(A)**, Grip strength (left, **(B)**, Walking pace **(C)**, and ALM **(D)**.

The IVW-MR analysis suggested significant negative associations between increased plasma Hcy levels and grip strength (right: effect = −0.036, SE = 0.032, *p* = 5.53E-4; left: effect = −0.045, SE = 0.010, *p* = 1.45E-5), walking pace (effect = −0.038, SE = 0.011, *p* = 3.18E-4), and ALM (effect = −0.058, 0.013, *p* = 1.03E-5), as shown in Table 3. Similar results were observed regarding the MR-Egger, weighted median, maximum likelihood, and penalized weighted median methods (Table 3). The direction of the results of all sensitive analysis methods is consistent with the direction of the IVW-MR results, suggesting that the results are stable. No significant heterogeneity was detected among the 7 IVs according to Cochran’s Q-test (Table 4); thus, all IVW-MR analyses used fixed-effects models. The MR‒Egger intercept test and MR-PRESSO global test indicated that the MR results were unlikely to be confounded by horizontal pleiotropy and reverse causality (Table 4). Therefore, all MR analyses included only the first round. The effect of individual SNPs on causal estimation was demonstrated by forest plots (Figure 1). The leave-one-out analysis showed that the MR analysis results were stable and unaffected by any single SNP (Figure 2).

**TABLE 3:** MR analysis of association between homocysteine levels and the three components of sarcopenia.

Traits	SNP(n)	Methods	Beta	SE	p Value
Hcy levels (unit increase)					
Grip strength (right)	7	MR-Egger	−0.013	0.032	6.90E–01
	7	Weighted_median	−0.036	0.013	6.02E–03
	7	IVW	−0.036	0.010	5.53E–04
	7	Maximum likelihood	−0.036	0.011	6.40E–04
	7	Penalised weighted median	−0.036	0.013	7.04E–03
Grip strength (left)	7	MR-Egger	−0.019	0.032	5.79E–01
	7	Weighted_median	−0.045	0.013	6.80E–04
	7	IVW	−0.045	0.010	1.45E–05
	7	Maximum likelihood	−0.046	0.011	2.05E–05
	7	Penalised weighted median	−0.045	0.014	1.24E–03
Walking pace	7	MR-Egger	−0.072	0.032	7.47E–02
	7	Weighted_median	−0.036	0.013	5.66E–03
	7	IVW	−0.038	0.011	3.18E–04
	7	Maximum likelihood	−0.038	0.011	3.60E–04
	7	Penalised weighted median	−0.036	0.013	6.36E–03
ALM	7	MR-Egger	−0.056	0.041	2.26E–01
	7	Weighted_median	−0.057	0.017	1.01E–03
	7	IVW	−0.058	0.013	1.03E–05
	7	Maximum likelihood	−0.059	0.014	1.55E–05
	7	Penalised weighted median	−0.057	0.017	8.39E–04
	Hcy levels (unit decrease)				
Grip strength (right)*	4	MR-Egger	0.008	0.071	0.93
	4	Weighted_median	0.013	0.016	0.42
	4	IVW	0.005	0.021	0.82
	4	Maximum likelihood	0.005	0.013	0.71
	4	Penalised weighted median	0.014	0.016	0.38
Grip strength (left)*	3	MR-Egger	0.064	0.052	0.43
	3	Weighted_median	0.004	0.016	0.81
	3	IVW	−0.006	0.014	0.64
	3	Maximum likelihood	−0.007	0.014	0.64
	3	Penalised weighted median	0.004	0.016	0.81
Walking pace	5	MR-Egger	0.031	0.066	0.67
	5	Weighted_median	0.014	0.016	0.39
	5	IVW	0.010	0.020	0.61
	5	Maximum likelihood	0.011	0.012	0.38
	5	Penalised weighted median	0.019	0.016	0.25
ALM*	4	MR-Egger	1.80E–4	0.080	0.99
	4	Weighted_median	−0.032	0.021	0.14
	4	IVW	−0.034	0.018	0.06
	4	Maximum likelihood	−0.034	0.018	0.06
	4	Penalised weighted median	−0.032	0.021	0.13

*: Second round of MR, analysis after identification and removal of pleiotropic SNPs, by MR-PRESSO; Of these, rs2251468 was excluded in the MR, analysis of grip strength (right), rs2251468 and rs42648 were excluded in the MR, analysis of grip strength (left), and rs7130284 was excluded in the MR, analysis of ALM.

**TABLE 4:** Heterogeneity and pleiotropy analysis between homocysteine levels and the three components of sarcopenia.

	Heterogeneity test				MR-egger intercept test					MR-PRESSO global test			
	Q	Q_df	P value	Q	Q_df	P value	Intercept	SE	P value	Pleiotropy variant #	P value	Pleiotropy variant *	P value
Hcy levels (unit increase)													
Grip strength (right)	3.60	6	0.73	3.05	5	0.69	−0.001	0.002	0.49	NA	0.81		
Grip strength (left)	5.76	6	0.45	5.03	5	0.41	−0.002	0.002	0.43	NA	0.57		
Walking pace	6.30	6	0.39	5.00	5	0.42	0.002	0.002	0.31	NA	0.44		
ALM	4.13	6	0.66	4.13	5	0.53	＜0.001	0.002	0.95	NA	0.74		
Hcy levels (unit decrease)													
Grip strength (right)	7.93	3	0.05	7.92	2	0.02	＜0.001	0.005	0.97	rs2251468	0.02	NA	0.13
Grip strength (left)	3.55	2	0.17	1.18	1	0.28	−0.006	0.004	0.39	rs2251468/rs42648	＜0.01	NA	NA
Walking pace	11.20	4	0.02	10.80	3	0.01	−0.001	0.004	0.76	NA	0.07		
ALM	0.55	3	0.91	0.36	2	0.84	−0.002	0.005	0.71	rs7130284	0.06	NA	0.89

#: The first round MR, analysis; *: The second round of MR, analysis after identification and removal of pleiotropic SNPs, by MR-PRESSO. Q: Cochran Q statistics; SE, standard error.

### 3.3 Associations of decreased plasma hcy levels with grip strength, walking pace, and appendicular lean mass

**FIGURE 3:**
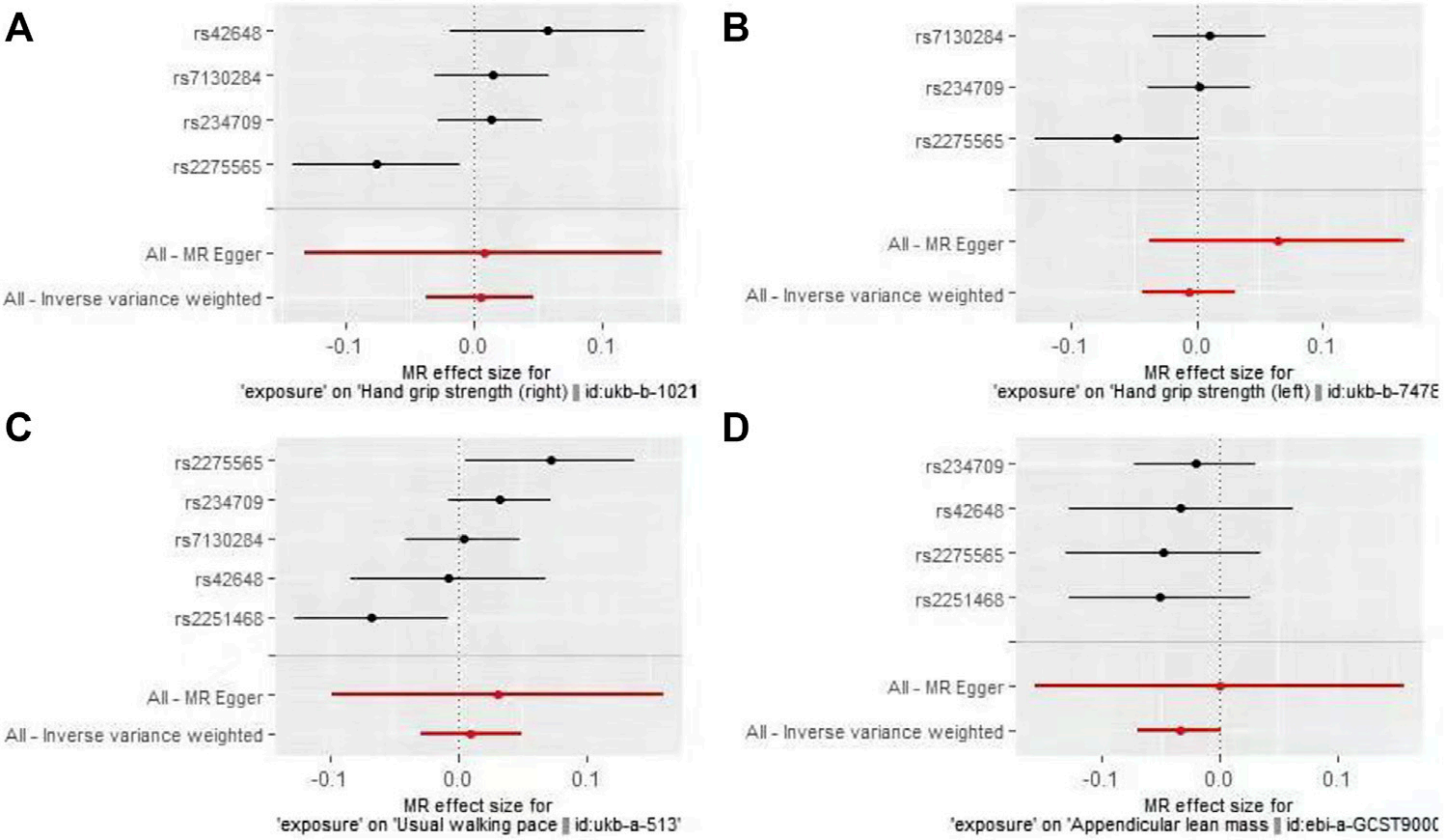
Forest plots for MR analyses of the causal effect of decreased plasma homocysteine levels on Grip strength (right, **(A)**, Grip strength (left, **(B)**, Walking pace **(C)**, and ALM **(D)**.

**FIGURE 4:**
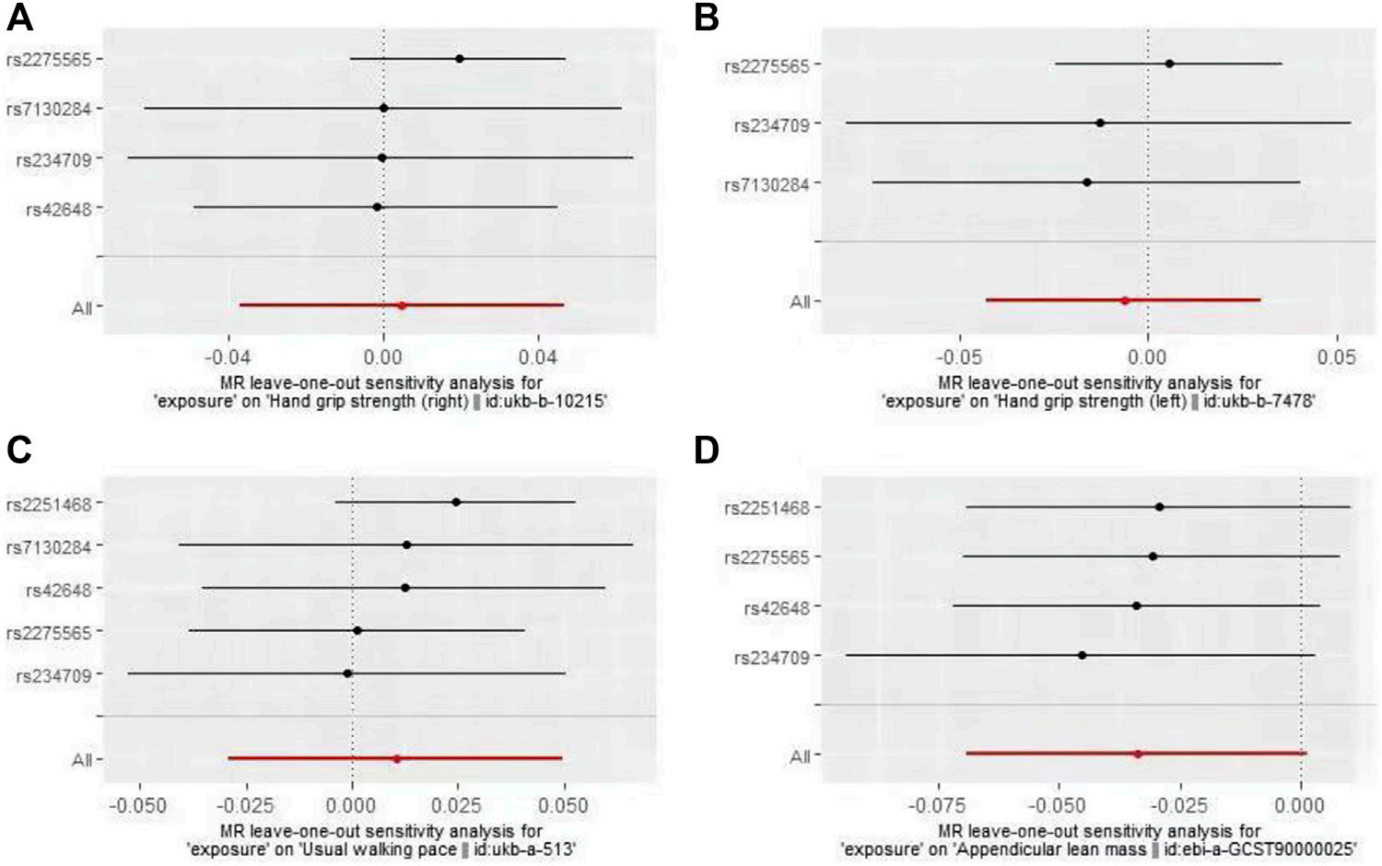
Plots of “leave-one-out”analyses MR analyses of the causal effect of decreased plasma homocysteine levels on Grip strength (right, **(A)**, Grip strength (left, **(B)**, Walking pace **(C)**, and ALM **(D)**.

In the first round of analysis, the MR-PRESSO outlier test identified four pleiotropic SNPs associated with grip strength (right: rs2251468; left: rs2251468 and rs42648) and ALM (rs7130284). Therefore, a second round of analysis was performed after excluding the pleiotropic SNPs. The IVW-MR analysis found no evidence of associations between decreased plasma Hcy levels and grip strength (right: effect = 0.005, SE = 0.021, *p* = 0.82; left: effect = −0.006, SE = 0.014, *p* = 0.64), walking pace (effect = 0.01, 0.020, *p* = 0.61), or ALM (effect = −0.034, SE = 0.018, *p* = 0.06), (Table 3; Figure 3). The results of all MR methods used for sensitivity analysis are shown in Table 3. In the MR analysis of grip strength (left), the results of MR-Egger, Weighted median and Penalised weighted median were opposite to those of IVW-MR; In the MR analysis of ALM, the result of MR-Egger was opposite to that of IVW-MR, but the results of all MR methods in grip strength (left) and ALM were not significant, suggesting that the results were also stable. In the MR analyses of grip strength (right) and walking pace, the results of the MR methods used for sensitivity analysis were in the same direction as the IVW-MR results. Cochran’s Q-test detected slight heterogeneity in IVs associated with grip strength (right) and walking pace (Table 4); therefore, the IVW-MR analysis utilized random-effects models. No heterogeneity was observed in IVs associated with grip strength (left) or ALM (Table 4); thus, the IVW-MR analysis utilized fixed-effects models. The MR‒Egger intercept test and MR-PRESSO global test indicated that the MR results were unlikely to be confounded by horizontal pleiotropy and reverse causality (Table 4). The effect of individual SNPs on causal estimation was demonstrated by forest plots (Figure 3). Additionally, the leave-one-out analysis showed that the MR analysis results were stable and unaffected by any single SNP (Figure 4).

## 4 Discussion

To our knowledge, this is the first study to use large-scale GWAS data and MR analysis to explore the causal relationship between Hcy levels and the three components (grip strength, walking pace and ALM) of sarcopenia. This MR study showed that increased plasma Hcy levels were significantly and negatively associated with grip strength, walking pace, and ALM, and similar results were obtained in all sensitivity analyses. However, decreased plasma Hcy levels were not associated with grip strength, walking pace, or ALM. Although inconsistent directions emerged in the sensitivity analysis of grip strength (left) and ALM, but since the results of all MR methods are not significant, we consider that this no-causality association is still the stable. Based on this, our findings provide new insights into the effects of Hcy on muscle function and mass.

Consensus regarding the relationship between plasma Hcy levels and muscle strength and physical performance in observational studies is lacking. Granic et al. (2017) did not find an association between Hcy levels and grip strength in a longitudinal study that included 845 participants aged 85 years or older, either in the cross-sectional study (wave 1) or in the subsequent 5-year follow-up (waves 2–4); however, the use of this elderly population (>85 years old) may reduce its generalizability to older individuals of less advanced age. In addition, Hcy levels in Granic et al.‘s study were measured by detecting Hcy biomarkers and further determined by principal component analysis (PCA); thus, the results may have been influenced by the selection and number of biomarkers included in the PCA(20). In contrast, a cross-sectional study by Lee et al. (2020) that included 1,582 community members >50 years of age reported significant negative associations of increased Hcy levels with grip strength and walking pace. These strong associations persisted after adjusting for confounding factors such as sex, age, smoking status and underlying disease. Conflicting results between observational studies may be explained by interference from confounding factors. In this MR study, to eliminate the interference of confounding factors (pleiotropic SNPs) on the results, we took a series of quality control steps for instrumental variable selection (PhenoScanner database checking and MR-PRESSO outlier test), then we found a significant negative correlation between high levels of Hcy with grip strength and walking pace, which may resolve the current controversy to some extent. Furthermore, several animal experiments have reported that increased Hcy levels influence skeletal muscle function, consistent with our findings. Using a mouse model of hyperhomocysteine (HHcy) constructed using cysteine beta synthase deficient (CBS-) mice, Veeranki et al. (2015) concluded that HHcy leads to reduced proliferation of skeletal muscle satellite cells by inducing excessive oxidative stress and p38 MAPK signaling. Another experiment utilizing a CBS- mouse model found that skeletal muscle weakness associated with HHcy involves mitochondrial dysfunction and epigenetic modifications (Majumder et al., 2018). Thus, some of the mechanisms by which increased plasma Hcy levels lead to skeletal muscle dysfunction have been identified by *in vivo* experiments. Physical performance is a multidimensional concept involving not only muscles but also central and peripheral nerve function. The pathogenesis of HHcy in various vascular conditions, such as induction of oxidative stress (Behera et al., 2021), promotion of apoptosis (Pai et al., 2021), and stimulation of inflammatory factor release (Liang et al., 2021), has been extensively investigated; similarly, studies are exploring the pathogenesis of HHcy in various neurological diseases (Lipton et al., 1997; Moretti and Caruso, 2019). Although it is unclear whether HHcy reduces physical performance through muscle damage or neurological damage, there is no doubt that Hcy impacts muscles, nerves, and even blood vessels and thereby leads accelerates declines in physical performance.

However, it remains controversial whether Hcy reduces muscle mass. Several previous studies (Elshorbagy et al., 2008; Atkins et al., 2014) did not find a significant association between HHcy and muscle mass. However, these studies were either biased in terms of sample selection (e.g., hospitalized participants rather than community-dwelling older adults) or did not control for confounding factors associated with sarcopenia or Hcy levels (e.g., lifestyle, underlying medical conditions, and mental health) to a great extent. Choi et al. (2022) conducted a cross-sectional study of 114,583 community-dwelling adults and found significant associations of Hcy levels with mild low muscle mass (LMM) and severe LMM. These associations persisted after adjusting for factors such as age, sex, lifestyle, physical activity, and glycated hemoglobin. In another study of 423 patients with peripheral arterial disease, a significant inverse association was found between plasma Hcy levels and calf muscle area (McDermott et al., 2007). Similarly, in the present MR study, we found a significant negative correlation between increased Hcy levels and ALM. This etiologically verified negative correlation suggests that the abovementioned pathophysiological mechanism by which Hcy damages muscle not only impairs muscle function but also reduces muscle mass. In the future, well-designed *in vivo* or animal experiments are needed to elucidate the exact mechanisms underlying Hcy-induced muscle damage.

The use of MR analysis is a major strength of this study, as it allowed the use of large-scale genome-wide summary data, thus improving the reliability of our findings. To minimize potential bias due to population stratification, the GWAS data used in this study were all sourced from European populations. We also performed a linkage disequilibrium test to exclude the effects of nonindependent SNPs on the results. To prevent the influences of pleiotropy and reverse causation, we first identified and excluded pleiotropic SNPs that were significantly associated with outcomes or confounders with the PhenoScanner database before the first round of analysis. As the exact biological functions of many genetic variants remain unknown, we also identified and removed potentially pleiotropic SNPs with the MR-PRESSO outlier test before the second round of analysis. Our MR study showed a causal correlation between Hcy levels and the major components of sarcopenia, so Hcy levels can be used as an early predictor of sarcopenia. Also, because of the causal association, reducing Hcy levels could also be used as a treatment for sarcopenia.

This study has some limitations. First, this study did not determine the relationship between Hcy levels and sarcopenia based on the cutoff values required for the diagnosis of sarcopenia. However, since grip strength, walking pace and ALM are valid predictors of sarcopenia (Cruz-Jentoft et al., 2019), we believe that this study supports a negative association between increased plasma Hcy levels and sarcopenia. Second, in our study, ALM was measured using BIA, an indirect measure that may be less accurate than other methods that directly quantify muscle mass, such as magnetic resonance imaging (MRI), computed tomography (CT) or dual-energy x-ray absorptiometry (DXA). However, the EWGSOP2 consensus repor (Cruz-Jentoft et al., 2019) states that, considering affordability and portability, BIA-based muscle mass measurements may be preferable to other measures in large populations. Third, physical performance can be measured by different tests such as walking pace, short distance physical performance test (SPPB) and timed up and go test (TUG). We only use walking pace as an assessment of physical performance, because it is considered a rapid, safe and highly reliable test for sarcopenia and has been shown to predict adverse outcomes associated with sarcopenia—disability, falls and death (Cruz-Jentoft et al., 2019). In addition, sex differences in skeletal muscle kinetics and fiber composition necessitate sex-specific analyses of muscle function and mass (De Giuseppe et al., 2022). Because we were unable to obtain sex-stratified GWAS data on plasma Hcy levels, we did not investigate the effect of Hcy levels on sarcopenia in a sex-stratified manner. Finally, these results may not be generalizable to populations of other ethnicities, as this study was primarily limited to individuals of European ancestry.

## 5 Conclusion

In conclusion, our MR analysis supported a causal association between increased plasma Hcy levels and lower grip strength, slower walking pace, and decreased ALM, while decreased plasma Hcy levels were not associated with all three components of sarcopenia. Prevention of increased plasma Hcy levels may avoid the occurrence of sarcopenia.

## Data Availability

Publicly available datasets were analyzed in this study. This data can be found here: See Table 1 for data sources and direct links.
