# Supplementary material for: Causal effects of homocysteine levels on the components of sarcopenia: A two-sample mendelian randomization study
Source: Front Genet. 2022 Nov 22;13:1051047. doi: 10.3389/fgene.2022.1051047 (PMC9722755; doi:10.3389/fgene.2022.1051047)
Supplement: Supplementary file 1 [file Presentation1.pdf]

Study/Cohort Characteristics

Rotterdam Study I (RSI) and Rotterdam Study II (RSII): The RS is a population-based cohort study aimed at assessing the occurrence of and risk factors for chronic diseases in the elderly [1]. For RSI, all inhabitants of Ommoord, a district of Rotterdam in the Netherlands, who were 55 years or older were invited and 7983 agreed (78% response rate) to participate. Baseline visits took place between 1990 and 1993. In 1999, inhabitants who turned 55 years of age or moved into the study district since the start of the study were invited (RSII) of whom 3011 participated (67% response rate). In total there were tHcy and genetic data available for 3414 (RSI) and 1868 (RSII) individuals respectively.

Women's Genome Health Study (WGHS): The Women's Genome Health Study (WGHS) is a prospective cohort of female, North American, health care professionals representing participants in the Women's Health Study who provided a blood sample at baseline and consent for blood-based analyses. Participants in the WHS were 45 or older at enrolment and free of cardiovascular disease, cancer or other major chronic illness [2].

Nurses' Health Study (NHS): The NHS was initiated in 1976, when 121,700 United States registered nurses between the age of 30 and 55, residing in 11 larger US states, returned an initial questionnaire reporting medical histories and baseline health-related exposures. Biennial questionnaires with collection of exposure

information on risk factors have been collected prospectively, and outcome data with follow-up of reported disease events are collected. From May 1989 through September 1990, we collected blood samples from 32,826 participants in the NHS cohort. Subsequent follow up has been greater than 99% for this sub-cohort.

The TwinsUK cohort: A registry of healthy, adult, female identical and non-identical twins, aged between 18 and 79 which have been previously shown to be representative of the general UK population [3]. Plasma homocysteine levels were measured in a total of 2,009 female twins of which 1172 had GWAS data available.

The CoLaus cohort: A population-based sample consisting of 6738 male and female, Caucasian residents of Lausanne (Switzerland) aged 35 to 75 years. The study design and protocol have been described in detail previously [4]. A total of 5,434 individuals with phenotype and WGS data were included in the meta-analysis.

Baltimore Longitudinal Study of Aging (BLSA): The Baltimore Longitudinal Study of Aging (BLSA) is an observational study that began in 1958 to investigate normative aging in community dwelling adults who were healthy at study entry (clinicaltrials.gov/ct2/show/NCT00233272).

Cardiovascular Health Study (CHS): The CHS is a population-based cohort study of risk factors for CHD and stroke in adults ≥65 years conducted across four field centers in the United States: Forsyth County, NC; Sacramento County, CA;

Washington County, MD; Pittsburgh, PA. [5] The original predominantly Caucasian cohort of 5,201 persons was recruited in 1989-1990 from random samples of the Medicare eligibility lists; subsequently, an additional predominantly African-American cohort of 687 persons was enrolled for a total sample of 5,888. DNA was extracted from blood samples drawn on all participants at their baseline examination in 1989-90. In 2007-2008, genotyping was performed at the General Clinical Research Center's Phenotyping/Genotyping Laboratory at Cedars-Sinai using the Illumina 370CNV BeadChip system on 3980 CHS participants who were free of CVD at baseline, consented to genetic testing, and had DNA available for genotyping. .

Framingham Heart Study (FHS):

The FHS began in 1948 with the enrolment of two-thirds of the adult population of Framingham, Massachusetts, including 2873 women aged 28–62 years. In 1971, 5124 offspring of the original cohort members and offspring spouses were enrolled in the Framingham Heart Study, including 2641 women ranging in age from 12 to 60 years.

Nijmegen Biomedical Study (NBS):

The Nijmegen Biomedical Study is a population-based survey conducted by the Radboud University Nijmegen Medical Centre [6]. From a total of 22,500 age and sex stratified inhabitants of Nijmegen that were randomly selected, 9,371 individuals participated. Blood and serum were collected from 6434 out of these 9,371

individuals. Subjects between 50-70 years old were invited for a second visit in which fasting plasma was obtained in 1517 subjects. In total, 550 samples with genome-wide SNP-array and imputed SNP data and plasma tHcy concentrations were available for the current study.
